# An Online Resource for Monitoring 24-Hour Activity in Children and Adolescents: Observational Analysis

**DOI:** 10.2196/59283

**Published:** 2024-11-18

**Authors:** Benny Kai Guo Loo, Siao Hui Toh, Fadzlynn Fadzully, Mohammad Ashik Zainuddin, Muhammad Alif Abu Bakar, Joanne Shumin Gao, Jing Chun Teo, Ethel Jie Kai Lim, Beron Wei Zhong Tan, Michael Yong Hwa Chia, Terence Buan Kiong Chua, Kok Hian Tan

**Affiliations:** 1 Sport and Exercise Medicine Service, KK Women’s and Children’s Hospital, 100 Bukit Timah Road, Singapore, 229899, Singapore, 65 63948488; 2 Paediatrics Academic Clinical Programme, SingHealth Duke-National University of Singapore Academic Medical Centre, Singapore, Singapore; 3 Physiotherapy Department, KK Women’s and Children’s Hospital, Singapore, Singapore; 4 Digital Integration Medical Innovation and Care Transformation, KK Women’s and Children’s Hospital, Singapore, Singapore; 5 Nutrition & Dietetics Department, KK Women’s and Children’s Hospital, Singapore, Singapore; 6 Psychology Service, KK Women’s and Children’s Hospital, Singapore, Singapore; 7 Physical Education & Sports Science, National Institute of Education, Nanyang Technological University, Singapore, Singapore; 8 Division of Obstetrics and Gynaecology, KK Women’s and Children’s Hospital, Singapore, Singapore

**Keywords:** online, physical activity, sedentary behaviour, sleep, diet, 24-hour activity, child, adolescent

## Abstract

**Background:**

The Singapore integrated 24-hour activity guide for children and adolescents was introduced to promote healthy lifestyle behaviors, including physical activity, sedentary behavior, sleep, and diet, to enhance metabolic health and prevent noncommunicable diseases. To support the dissemination and implementation of these recommendations, a user-friendly online resource was created to help children and adolescents adopt these behaviors in Singapore.

**Objective:**

This study aimed to assess the acceptability of the online resource in the adoption of healthier lifestyle behaviors, and the change in the users’ behaviors with the use of this online resource.

**Methods:**

Participants aged 7-17 years were required to log their activity levels of the past 7 days at the beginning and at the end of a 3-month period using the browser-based online resource, including information on the duration and frequency of moderate- to vigorous-intensity physical activity (MVPA), length of sedentary behavior, duration and regularity of sleep, and food portions. User satisfaction, on the length, ease of use, and relevance of the online resource, was also recorded using a 10-point Likert scale. Descriptive statistics and statistical analyses, including the Wilcoxon signed rank test and McNemar test, were carried out at baseline and at the end of 3 months.

**Results:**

A total of 46 participants were included for analysis. For physical activity, the number of days of MVPA increased from a median of 3 (IQR 2‐5) days to 4 (IQR 2‐5) days (*P=*.01). For sedentary behavior, the median daily average screen time decreased from 106 (IQR 60‐142.5) minutes to 90 (IQR 60‐185) minutes. For sleep, 10% (5/46) more participants met the recommended duration, and the number of days with regular sleep increased from a median of 6 (IQR 5‐7) days to 7 (IQR 5‐7) days (*P=*.03). For diet, there was a decrease in the portion of carbohydrates consumed from a median of 42% (IQR 30‐50) to 40% (IQR 30‐48.5; *P=*.03), and the number of days of water and unsweetened beverage consumption remained stable at a median of 5 days but with a higher IQR of 4‐7 days (*P=*.04). About 90% (39-41/46) of the participants reported that the online resource was relevant and easy to use, and the rating for user satisfaction remained favorable at a median of 8 with a higher IQR of 7‐9 (*P=*.005).

**Conclusions:**

The findings support the development of a dedicated online resource to assist the implementation of healthy lifestyle behaviors based on the Singapore integrated 24-hour activity guide for children and adolescents. This resource received favorable ratings and its use showed the adoption of healthier behaviors, including increased physical activity and sleep, as well as decreased sedentary time and carbohydrate consumption, at the end of a 3-month period.

## Introduction

The World Health Organization (WHO) has identified the adoption of healthy lifestyle behaviors as a key preventive strategy against noncommunicable diseases (NCDs), such as cardiovascular diseases, cancers, and diabetes [1]. This strategy is best implemented when one is young, as children are not immune to the adverse health effects of suboptimal lifestyle behaviors [1]. Local longitudinal studies have also shown that children suffer adverse health effects from suboptimal lifestyle behaviors, including higher BMI from a lack of physical activity [2], longer screen time [3,4], and insufficient sleep [5]. Shorter sleep was also associated with decreased body length in the first 2 years of life [5]. This is even more concerning given the rise in the percentage of local overweight school-going children from 11% in 2013 to 17% in 2021 [6], and that less than half of these children are achieving the recommended amount of physical activity [7].

To improve the metabolic health of Singaporean children and adolescents aged 7-17 years, Singapore integrated 24-hour activity guide for children and adolescents was introduced to provide up-to-date and evidence-based recommendations on physical activity, sedentary behaviors, sleep and eating habits, framed within a 24-hour period [7]. During the development of this guide, Quah et al [8] surveyed 100 parents of school-going children revealing that they were concerned about the lack of physical activity and excessive screen-viewing time in their children, but only about half of these parents were aware of what the recommendations are for these activities. The findings from this study also highlight the importance of dissemination and implementation of the guide, including the development of resources to improve the awareness and adoption of this guide in the community [8].

Therefore, the aim of this pilot study was to create a user-friendly online resource to assess a child’s or adolescent’s activity levels based on the guide, with advice on improving or maintenance of the respective behaviors. The objectives were to assess the acceptability of the online resource in the adoption of healthier lifestyle behaviors, and the change in the user’s behaviors with the use of this online resource.

## Methods

### Study Sample

The participants were recruited using a convenience sampling method via posters and social media advertisements. Eligible participants had to be of school-going age from 7 to 17 years, have access to electronic devices with an internet connection, and be able to participate in physical activity. Participants were required to log their activity levels using the online resource at the beginning and the end of a 3-month period (ie, baseline entry during the first week and final entry during the 12th week of the 3-month period).

### Ethical Considerations

This study obtained ethics approval with an exempt review from the SingHealth Centralised Institutional Review Board (reference number 2021/2403). Informed consent from parents and assent from participants were obtained before using the online resource, and they were allowed to withdraw from the study at any time. All data were de-identified using unique study IDs. All participants received SGD 20 (US $15.07) upon completing each survey as compensation for their time.

### Resource Design and Data Collection

**Figure 1.**
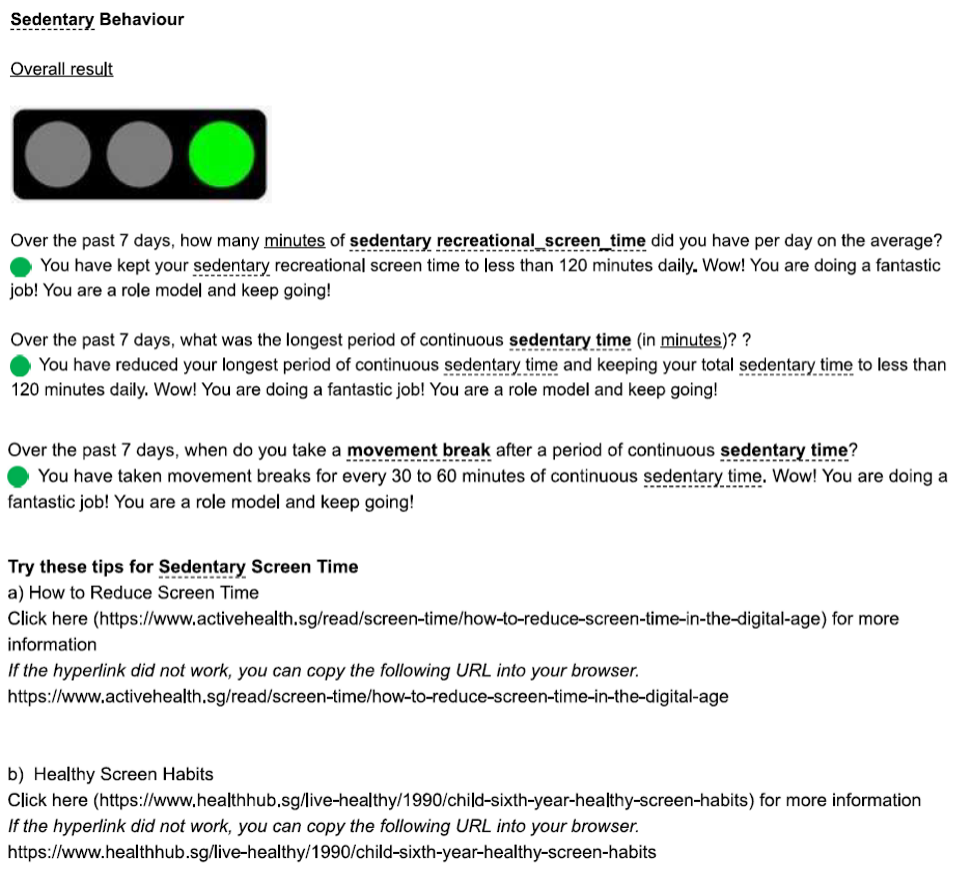
Sample of report, messages and advice generated in the online resource tool (green color shown) when recommendations are met for sedentary behavior.

**Figure 2.**
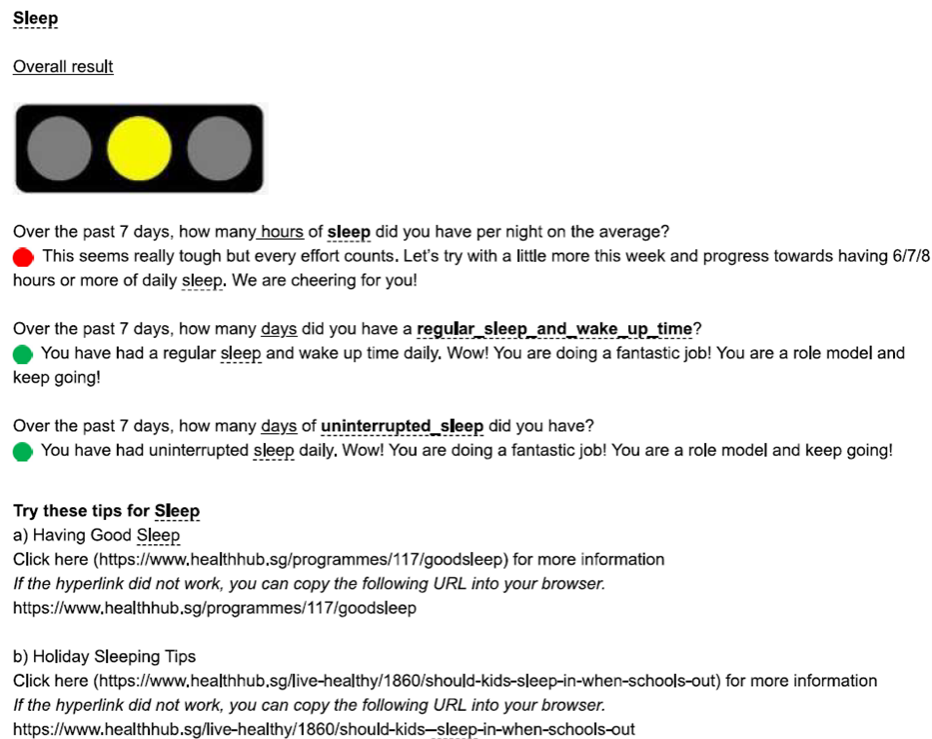
Sample of report, messages, and advice generated in the online resource tool (yellow color shown) when some recommendations are met for sleep.

**Figure 3.**
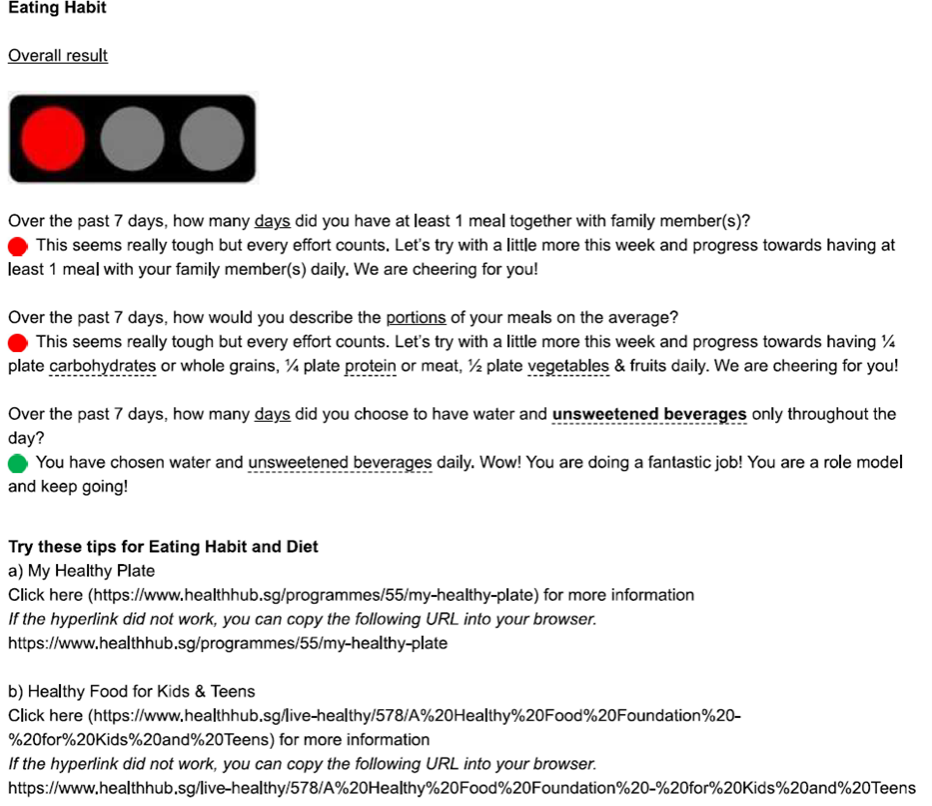
Sample of report, messages, and advice generated in the online resource tool (red color shown) when recommendations are not met for eating habits.

The online resource was designed as a browser-based activity diary. This resource was accessed via both computer and mobile devices. Each participant was assigned a unique username and a random password was generated for each entry. To improve the user interface and to minimize incomplete or missing data, participants were required to key in their information or activity level using a drop-down list or along a numerical bar. The questions for each activity domain were presented on a single page and were in sequential order (ie, all questions for physical activity were on the first page and all questions for sedentary behavior were on the next page). After entering all required data, the participant proceeded to the report page and was prevented from returning to edit the entered data. The resource was programmed to generate a report based on the data for each activity domain in the form of traffic light colors. Green was shown when the participant had met all recommendations for that domain, yellow if he or she met some, and red if none of the recommendations was achieved. Following the report, messages of encouragement and advice were displayed to help the participants maintain or improve their lifestyle behaviors (refer to Figures 1-3 for samples of reports, messages, and advice generated). Several links to recommendations and resources including the Singapore integrated 24-hour activity guide were also provided [7].

### Question Design and Content Validation

A total of 27 questionnaire items garnered data on demographics, physical activity, sedentary behavior, sleep, eating habits, and user satisfaction. Participants aged 9 years and younger were recommended to enter the data together with their parents, and those older than 9 years did by themselves and sought their parent’s guidance as needed. The participants were required to answer the activity questions based on their lifestyle activities over the past 7 days. Demographic information was solicited only when the participants used the resource for the first time, which included age, gender, ethnicity, height, and weight. The level of motivation for exercise of the participants was assessed using 4 questions adapted from Exercise Is Medicine, Australia [9]. In total, 3 questions were created for each activity domain based on the recommendations stated in the Singapore integrated 24-hour activity guide for children and adolescents. As there was no prior resource tool developed on the basis of this guide, all questions were created de novo by the guide developers. To improve the understanding and acceptability by local participants, the questions were written using similar language and styles used in the guide, and local resources were quoted whenever applicable. These questions were content validated qualitatively and quantitatively (content validity index of 0.94) for relevance and representativeness by two independent content experts using the response process validation procedure–an advocated best practice procedure outlined by Yusoff [10]. The content validity index of 0.94 is within the recommended acceptable range of at least 0.80 [10].

### Assessment of Physical Activity and Sedentary Behavior

**Figure 4.**
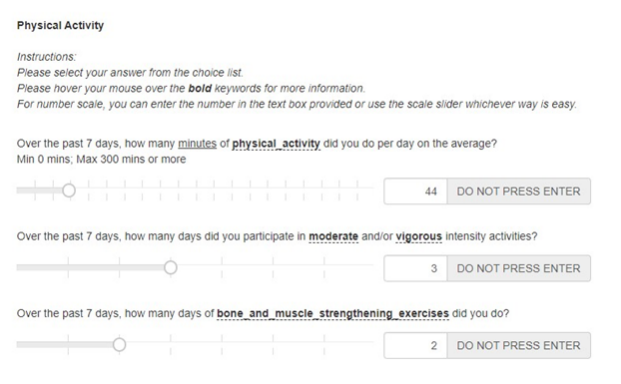
Questions asked and sample response in the online resource tool for physical activity.

To assess the quantity of physical activity participation in the past 7 days, the participants were asked about the number of minutes of physical activity (inclusion of all intensities) engaged per day on average, the number of days they participated in activities of moderate to vigorous intensity, and the number of days they participated in muscle- and bone-strengthening exercises (refer to Figure 4). The recommended amount of physical activity is to engage in 420 minutes or more of moderate- to vigorous-intensity physical activity (MVPA) and to participate in 3 or more days of muscle- and bone-strengthening activities in a week [7].

To assess the amount of sedentary behavior over the past 7 days, the participants were asked the daily average of sedentary recreational screen time, the longest period of continuous sedentary time, and when they took a movement break after a period of sedentary time. All these periods were participant-reported in minutes. The recommended average amount of screen time and continuous sedentary time is 120 minutes or less, and the recommended movement break is after every 60 minutes or less of sedentary time [7].

### Assessment of Sleep and Eating Habits

To assess the quantity and quality of their sleep in the past 7 days, the participants were asked the average number of hours of sleep per night, the number of days they had regular sleep, and the number of times they woke up from sleep as well as the number of days of uninterrupted sleep. The recommended duration of sleep is according to the participant’s age group (9-11 h for 7-13 y and 8-10 h for 14-17 y), and they are recommended to have regular and uninterrupted sleep daily (ie, 7 d per week) [7].

**Figure 5.**
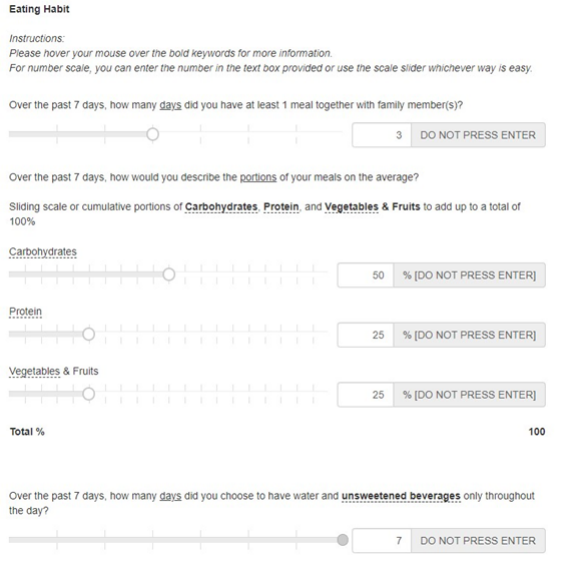
Questions asked and sample response in the online resource tool for eating habits.

To assess the characteristics of their eating habits over the past 7 days, the participants were asked the number of days they had at least one meal with their family members, the average portions of carbohydrate, protein, vegetable and fruit consumed per meal (adding to a total of 100%), and the number of days they drank water and unsweetened beverages (refer to Figure 5). The recommended portion of food is 25% of carbohydrate, 25% of protein, and 50% vegetables and fruit, and it is recommended to have at least one meal with their family members and to choose water and unsweetened beverages daily (ie, 7 d per week) [7].

### User Satisfaction

The participants were asked about their opinion of the length of the survey (too short, just right, or too long), if the questions were easy to understand and if the online resource was easy to use (yes or no), if they thought that the questions were relevant to their health (important, somewhat important, not sure, somewhat not important, or not important), and their user satisfaction of the online resource using a 10-point Likert scale rating (1=very poor; 10=very good).

### Statistical Analyses

Data analyses were conducted using the IBM SPSS Statistics for Windows (version 28.0). Continuous variables were presented as median and IQR and categorical variables as numbers and percentages. The Wilcoxon signed rank test and McNemar test were used to compare continuous variables and categorical variables, respectively, between the baseline and 3-month follow-up responses. Statistically significant results were determined at two-sided *P* values of less than *.*05. Effect size was measured using probability superiority with a value of more than 0.5 indicating a large effect for the 3-month follow-up assessments when compared with baseline assessments.

## Results

### Study Participants

A total of 57 participants were recruited from July to December 2022 and 11 participants were excluded due to incomplete data entry (ie, N=46). The details of the participants included are presented in Table 1. The mean age of the participants was 9.9 (SD 2.6) years with 52% (24/46) males, and the majority being Chinese (40/46, 87%). Most of the participants (37/46, 81%) reported that they have been physically active for the past 6 months (ie, maintenance phase in the 5 stages of change), and 9% (4/46) in the preparation phase with the remaining in the contemplation or precontemplation phase of exercise motivation [9].

**Table 1.** Characteristics of participants including their level of motivation (N=46).

Characteristics		Included participants (N=46)
Age (years), mean (SD)		9.9 (2.6)
**Sex, n (%)**		
	Male	24 (52)
	Female	22 (48)
**Ethnicity, n (%)**		
	Chinese	40 (87)
	Others	6 (13)
**Exercise motivation level, n (%)**		
	Maintenance	37 (81)
	Action	0 (0)
	Preparation	4 (9)
	Contemplation	2 (4)
	Precontemplation	3 (7)

### Survey Satisfaction

A survey of participants’ first-time usage of the online resource showed that 89% (41/46) agreed that the survey length was just right, and 85% (39/46) found the online resource easy to use. Almost 90% (41/46) of the participants reported that the questions were at least somewhat important to their health and 94% (43/46) found the questions easy to understand. The user satisfaction rating of the online resource was favorable at a median of 8 (IQR 6‐8), based on a 10-point Likert scale. The results of the participants’ 3-month follow-up rating showed that all aspects remained consistent and the rating was still favorable at a median of 8 with a higher IQR of 7‐9 (*P=*.005). The survey rating was statistically significant (refer to Table 2).

**Table 2.** Rating of online resource tool at baseline and 3-month follow-up.

Domain		Baseline	3-month follow-up	*P* value^a^
**Length of survey, n (%)**				
	Too short	1 (2)	0 (0)	.56
	Just right	41 (89)	42 (91)	
	Too long	4 (9)	4 (9)	
**Easy to understand, n (%)**				
	Yes	43 (94)	43 (94)	>.99
	No	3 (7)	3 (7)	
**Easy to use, n (%)**				
	Yes	39 (85)	43 (94)	.12
	No	7 (15)	3 (7)	
**Questions that are relevant to health, n (%)**				
	Important	20 (44)	23 (50)	.53
	Somewhat important	21 (46)	17 (37)	
	Not sure	1 (2)	4 (9)	
	Somewhat not important	4 (9)	2 (4)	
	Not important	0 (0)	0 (0)	
**User satisfaction rating of tool, median (IQR)**				
	Median rating	8 (6‐8)	8 (7‐9)	.005

a *P* values are calculated using Wilcoxon signed rank test and McNemar test for continuous variables and categorical variables, respectively.

### Baseline Activity Assessment

The duration of physical activity per day (for the past 7 d) recorded was a median of 60 (IQR 30‐120) minutes, and the number of days of MVPA (for the past 7 d) and muscle- and bone-strengthening activities (for the past 7 d) were a median of 3 (IQR 2.0‐5.0) days and 2 (IQR 1‐3) days, respectively. The daily sedentary screen time per day (for the past 7 d) recorded was a median of 106 (IQR 60‐142.5) minutes, and 21.8% (10/46) reported that their longest period of continuous sedentary behavior was above 120 minutes. The median period of daily sedentary behavior when the participants would take a movement break was 52.5 (IQR 30‐60) minutes.

In terms of sleep, the number of hours slept per day (for past 7 d) was a median of 8 (IQR 8‐9) hours, with 36.9% (17/46) meeting the recommended duration. The number of days with regular and uninterrupted sleep (for past 7 d) was a median of 6 (IQR 5‐7) days and 7 (IQR 5‐7) days, respectively. Just over 80% (37/46) of the participants reported having meals with their family daily over the past 7 days, and the median portions of carbohydrate, protein, and vegetable and fruit were 42% (IQR 30‐50), 30% (IQR 28‐35), and 30% (SD 20‐32.8), respectively. The number of days of drinking water and unsweetened beverages in the past 7 days was a median of 5 (IQR 1‐6) days with 20% (9/46) of the participants reporting doing so daily.

### 3-Month Follow-Up Activity Assessment

In a pre-to-post context, the average daily physical activity for the past 7 days remained stable at a median of 60 minutes but with a smaller IQR of 30‐62.5 minutes (*P=*.03), while the number of days of MVPA and muscle- and bone-strengthening activity increased to a median of 4 (IQR 2‐5.3) days (*P=*.01) and 3 (IQR 1‐5) days (*P=*.02), respectively. There was a decrease in the daily average sedentary screen time to a median of 90 (IQR 60‐185) minutes (*P=*.54), and fewer participants (14.3%, 7/46) reported their longest continuous period of sedentary time was more than 120 minutes. The median period of sedentary time before the participants took a movement break was also reduced to 30 (IQR 18.8‐60) minutes (*P=*.08). The changes in the average physical activity duration, number of days of MVPA, and number of days of muscle- and bone-strengthening exercises were statistically significant (refer to Table 3).

**Table 3.** Level of physical activity, sedentary behavior, sleep and eating habits over the past 7 days–baseline and 3-month follow-up assessments using the online resource tool.

Domain		Baseline	3-month follow-up	*P* value	Probability superiority
**Physical activity, median (IQR)**					
	Daily average amount (min)	60 (30-120)	60 (30-62.5)	.03	0.40
	Moderate to vigorous physical activity (days)	3 (2‐5)	4 (2‐5.3)	.01	0.66
	Muscle- and bone-strengthening activity (days)	2 (1‐3)	60 (30‐62.5)	.02	0.70
**Sedentary behavior (min), median (IQR)**					
	Daily average screen time	106 (60‐142.5)	90 (60‐185)	.54	0.58
	Longest continuous period	90 (60‐120)	90 (60‐120)	.79	0.52
	Period of sedentary time before taking a break	52.5 (30‐60)	30 (18.8‐60)	.08	0.58
**Sleep, median (IQR)**					
	Daily average amount (h)	8 (8‐9)	8 (8‐9)	.003	0.63
	Regular sleep (days)	6 (5‐7)	7 (5‐7)	.03	0.60
	Uninterrupted sleep (days)	7 (5‐7)	7 (6‐7)	.02	0.59
**Eating habits, median (IQR)**					
	Meals with family members (days)	7 (0)	7 (0)	.34	0.48
	Average portion of carbohydrate (%)	41.5 (30‐50)	40 (30‐48.5)	.03	0.64
	Average portion of protein (%)	30 (28‐35)	30 (28.8‐36.3)	.22	0.40
	Average portion of vegetable (%)	30 (20‐32.8)	30 (20‐35.3)	.10	0.62
	Water and unsweetened beverages (days)	5 (1‐6)	5 (4‐7)	.04	0.66

The sleep duration remained stable at a median of 8 (IQR 8‐9) hours (*P=*.003) but the increase in the minimum number of hours from 5 to 6 hours and the mean from 8.09 (SD 1.1) to 8.46 (SD 1.0) hours (data not shown) likely accounted for the significant *P* value. The percentage of participants meeting the recommended duration also increased to 48% (22/46; data not shown). The number of days with regular sleep increased to a median of 7 (IQR 5‐7) days (*P=.*03) while the number of days with uninterrupted sleep improved to a median of 7 (IQR 6‐7) days (*P=*.02). In terms of diet, there was a decrease in the percentage of participants having meals with their family daily (36/46, 78%). The median proportion of carbohydrate decreased to 40% (IQR 30‐48.5*; P=.*03), whereas the proportions of protein and fruit and vegetable remained stable at 30% (IQR 28.8‐36.3*; P=.*22) and 30 (IQR 20‐35.3*; P=.*10), respectively. The number of days of water and unsweetened beverage consumption also remained stable at a median of 5 days but with a higher IQR of 4‐7 days (*P=*.04). The average duration of sleep, number of days of regular sleep, number of days of uninterrupted sleep, portions of carbohydrate, and number of days of water and unsweetened beverage consumption were statistically significant (refer to Table 3).

## Discussion

### Principal Findings

This pilot study demonstrated that the novel online resource for improving lifestyle behaviors received consistent favorable ratings from the participants, with 87% to 89% agreeing that it was relevant to their health, and 85% to 94% agreeing that it was easy to use. These results help to reinforce the usefulness of online resources in assisting lifestyle behavioral changes and these may be in the form of a web-based tool [11,12], mobile app [13], or a combination of both [14]. van der Weegen et al [14] demonstrated that incorporating mobile and web-based tools in clinical practice increased the amount of participants’ physical activity and the effect was still evident after 3 months. Online dietary tools were also developed in the United States and the United Kingdom as a part of their strategy to intervene in dietary habits at the population level. However, most of these tools focus only on a single lifestyle behavior and there is a paucity of available online resources that support the contemporary 24-hour activity approach. Therefore, it is imperative to develop an online resource to support the Singapore integrated 24-hour activity guide for children and adolescents as a concerted effort to improve the lifestyle behaviors in this pediatric population.

The assessment of the participants’ activities also revealed that a significant proportion was unable to meet the recommendations in each activity domain. For instance, the calculated duration of MVPA in a week was 180 minutes (median average physical activity duration multiplied by median MVPA days – 60 min multiplied by 3 d), which was less than the recommended 420 minutes per week. Similarly, 63% (29/46) of the participants did not achieve the recommended sleep duration, and yet, the median sedentary screen time was close to 120 (IQR 60-142.5) minutes. Although the current recommendation on sedentary screen time is to be as low as possible, many parents might still be familiar with the previous recommendation of less than 120 minutes per day by the American Academy of Pediatrics [15]. In terms of their diet, the mean proportions of the main food groups also fell short of the recommended 25% of carbohydrate and protein, respectively, and 50% vegetable and fruit. These findings are consistent with studies in recent years, highlighting similar concerns that many children and adolescents in Singapore are not meeting these recommendations and hence not adopting healthy lifestyle habits [8,16,17]. Therefore, there is an ongoing need to educate the public on these recommendations and provide resources to aid them to adopt these lifestyle behaviors more easily.

Improvements in most lifestyle behaviors were observed when comparing the participants’ 3-month follow-up activity assessments with their baseline assessments. In terms of physical activity, the number of days engaging in MVPA and muscle- and bone-strengthening activities increased from a median of 3 (IQR 2.0-5.0) days and 2 (IQR 1.0-3.0) days to 4 (IQR 2.0-5.3) days and 3 (IQR 1.0-5.0) days, respectively. Consequently, the calculated median duration of MVPA for the week also increased to 240 minutes. The average sedentary screen time decreased from a median of 106 (IQR 60.0-142.5) minutes to 90 (IQR 60.0-185.0) minutes, and the participants were taking earlier movement breaks after a median sedentary period of 30 (IQR 18.8-60.0) minutes as compared with the initial 52.5 (IQR 30.0-60.0) minutes. There were also improvements seen in sleep and dietary habits. The number of days with regular sleep increased from a median of 6 (IQR 5.0-7.0) days to 7 (IQR 5.0-7.0) days, and their carbohydrate intake decreased from a median of 41.5% (IQR 30.0%-50.0%) to 40% (30.0%-48.5%). Other components showed consistent results, such as the average daily amount of physical activity, the daily average amount of sleep, and the number of days consuming water and unsweetened beverages when compared with their baseline assessments; no deteriorating trend was found. These results showed the promising potential of using the online resource to tackle multiple lifestyle behavior domains of a subgroup within a population, and the effects could be enhanced by combining its use with clinical services and extending its adoption over a longer period [14].

The current movement guidelines toward an integrated 24-hour activity approach are built upon the evidence that similar health benefits can be achieved by meeting the recommendations of various combinations of activity domains [18,19]. Children and adolescents who meet all the recommendations have been shown to receive the best health benefits [20,21]. With the inclusion of dietary recommendations in the Singapore integrated 24-hour activity guide, a more comprehensive and holistic behavioral guidance approach of eating, moving, sleeping, and sitting is adopted. At the start of the pilot study, only 1 participant reported meeting the 3 movement recommendations (ie, physical activity, sedentary behavior, and sleep) but none met all recommendations including the dietary ones. These data improved by the end of the 3-month pilot study with 5 participants meeting the 3 movement recommendations and 2 meeting all, including the dietary recommendations. This improvement was likely because of improved participant and parental awareness of current recommendations and monitoring of their activities.

The next phase includes expanding this online resource to other age groups and developing it as a mobile app to improve user accessibility and community penetration. Leveraging on the experience of Singapore Paediatric Activity–Related Evaluation (SPARE) tool development, current team members have joined another work group to develop a similar digital resource for children aged 0 to 2 years. The eventual aim is to integrate all these resources into the institution’s mobile health app so that a wider community can access and use these resources for themselves or their children.

### Strengths and Limitations

This study is the first pilot on a single online resource that integrated multiple lifestyle behavior domains based on the latest 24-hour recommendations for Singaporean children and adolescents. The strength of the study lies in the assimilation of multiple lifestyle behavior activities in a single resource, and with the survey questions adapted to the local context. The limitations of the study include selection bias from the convenience sampling method and recall bias as participants were required to report their past activities. The decision for a 7-day recall was because it is a standard duration to capture habitual behaviors across weekdays and weekends. The small sample size and short timeframe of this study limit the generalizability of the findings to the national population and observations of long-term behavioral changes. Another limitation is the expectancy effects with self-reporting measures, which may influence participants’ reporting toward socially acceptable norms.

### Conclusions

This study showed that there were consistent favorable ratings from participants on its use. There was also the adoption of healthier lifestyle behaviors when comparing the participants’ 3-month follow-up activity assessments with their baseline assessments after using the online resource, including increased physical activity and sleep while decreased sedentary time and carbohydrate consumption. The findings from this pilot study therefore support the role of developing a dedicated resource for the Singapore integrated 24-hour activity guide to assist in the promotion and implementation of the guide in children and adolescents [8]. Building a single resource catering for all activities provided easy access and likely catalyzed the awareness and adoption of these recommendations. The next phase would be to scale up the online resource in order to provide more convenient access, such as a mobile app, and to incorporate it into daily clinical practice.

## Abbreviations

MVPA: moderate- to vigorous-intensity physical activity
NCDs: non-communicable disease
SPARE: Singapore Paediatric Activity–Related Evaluation
WHO: World Health Organization
